# Detection of Salmonella Typhi in Bile by Quantitative Real-Time PCR

**DOI:** 10.1128/spectrum.00249-22

**Published:** 2022-05-31

**Authors:** Ellen E. Higginson, Joseph Nkeze, Jasnehta Permala-Booth, Irene N. Kasumba, Rosanna Lagos, Juan Carlos Hormazabal, Alexander Byrne, Gad Frankel, Myron M. Levine, Sharon M. Tennant

**Affiliations:** a Center for Vaccine Development and Global Health, University of Maryland School of Medicine, Baltimore, Maryland, USA; b Department of Medicine, University of Maryland School of Medicine, Baltimore, Maryland, USA; c Department of Pediatrics, University of Maryland School of Medicine, Baltimore, Maryland, USA; d Centro de Vacunas en Desarollo, Hospital de Ninos Roberto del Rio, Santiago, Chile; e Instituto de Salud Publica de Chile, Santiago, Chile; f MRC Centre for Molecular Bacteriology and Infection, Department of Life Sciences, Imperial College London, London, United Kingdom; Johns Hopkins Hospital

**Keywords:** bile, gall bladder, carrier, detection, typhoid

## Abstract

In countries where the incidence of typhoid fever is high, fecal material from short-term carriers of Salmonella Typhi contaminates inadequately treated water supplies. As treated water supplies and improved sanitation become available, chronic (mainly gallbladder) carriers of S. Typhi become important. The objective of this study was to develop a method for detection of S. Typhi in bile by quantitative real-time PCR (qPCR) in patients undergoing cholecystectomy. We evaluated sensitivity and specificity of probesets that target *oriC*, *viaB*, *fliC*-d, STY0201, and *stoD*. We optimized DNA extraction from bile and compared the sensitivity of culture and our qPCR method to detect S. Typhi in bile samples containing various cephalosporins. With the use of an optimized DNA extraction technique, our limit of detection of S. Typhi in spiked human bile samples was 7.4 × 10^2^ CFU/mL. We observed that S. Typhi could be detected by qPCR in samples containing cefazolin, cefotaxime, or ceftriaxone whereas culture could only detect Typhi in samples containing cefazolin but not cefotaxime or ceftriaxone. Our qPCR detection method for S. Typhi in bile should be preferred in areas where antibiotic usage is common.

**IMPORTANCE** New Salmonella Typhi conjugate vaccines have been deployed, which will potentially lead to a fall in incidence rates of typhoid fever in endemic areas. Identification of chronic carriers of S. Typhi will be important as these individuals can be a potential source of transmission to susceptible persons. To address this public health concern, we have developed a novel method to detect S. Typhi in bile using real-time PCR. Our method can be used to identify carriers of S. Typhi among patients undergoing cholecystectomy (gallbladder removal surgery). The sensitivity of our molecular-based assay was superior to culture when performed in the presence of antibiotics commonly used during surgery. Our methodology will complement efforts to eliminate typhoid disease.

## INTRODUCTION

Typhoid fever, caused by Salmonella enterica serovar Typhi (S. Typhi), remains endemic in many areas of the world. In addition to causing acute disease, this bacterium has the ability to cause chronic (>12 months) infection of the gallbladder in 3–5% of individuals following acute clinical or subclinical infections (1). Fecal contamination of foods prepared by such chronic carriers that are consumed by susceptible persons can result in new cases of typhoid fever, as exemplified by “Typhoid Mary” (2).

Most chronic gallbladder carriers have S. Typhi embedded within biofilms present on the surface of infected cholesterol gallstones (3). Surprisingly, little is known about the population-based prevalence of chronic carriers of S. Typhi in areas where typhoid is currently endemic or was so in the relatively recent past. However, two classic large studies have been carried out to measure the prevalence of presumed chronic carriage by bacteriologic isolation of S. Typhi from bile, gallstones, or gallbladder tissue of patients undergoing cholecystectomy in typhoid endemic areas. In the first study performed in 1980 on 1,000 consecutive patients in Santiago, Chile undergoing cholecystectomy (4), S. Typhi was isolated from 38 cholecystectomized patients (3.8%) and *S.* Paratyphi A or B from 35 (3.5%); thus, overall a typhoidal Salmonella serovar was isolated from 7.3% of the 1,000 patients. Uniquely, in Santiago there were robust data on the age-specific and gender-specific prevalence of cholelithiasis in the population. Thus, in conjunction with census data, it was possible to calculate that the population-based prevalence of putative chronic S. Typhi carriers was 694 per 10^5^ population above 10 years of age (5). In Santiago, as elsewhere, chronic typhoid carriage was more common in women and increased with age. In a more recent study on the prevalence of presumed chronic typhoid carriage in Kathmandu, Nepal, investigators cultured S. Typhi (*n* = 24) from 1,377 patients undergoing cholecystectomy (6). Thus, the prevalence of positivity in Kathmandu (3.3%) was approximately one-half that observed in Santiago. Since the prevalence of cholelithiasis in Kathmandu by age and sex was not known, it was not possible to estimate the population-based prevalence of chronic S. Typhi carriers.

In some typhoid endemic regions, investigators may seek to culture bile collected via upper intestinal sampling or from cholecystectomized patients to determine the prevalence of putative chronic gallbladder carriage of S. Typhi. Although culture continues to be the gold standard diagnostic, the administration of intravenous antibiotics immediately prior to cholecystectomy is now the standard of surgical care globally, which complicates the recovery of typhoidal Salmonella. Accordingly, to make cholecystectomy studies more effective and to help identify S. Typhi in bile or bile-containing duodenal fluid specimens from persons epidemiologically incriminated as putative contaminators of food vehicles in occasional outbreaks in industrialized countries, we undertook to design a quantitative real-time PCR (qPCR) diagnostic that could be used to specifically detect S. Typhi directly from bile.

Several studies have described qPCR diagnostic probesets for detection of S. Typhi (7–9). The goal of this study was to compare two of these probesets (targets STY0201 [*staG*] and *fliC*-d) with regard to their ability to detect S. Typhi in bile, as well as validate two novel probesets. In addition to identifying new qPCR diagnostic targets, we aimed to optimize bacterial DNA extraction from bile samples and to identify theoretical limits of detection for S. Typhi carriage in bile under optimized qPCR conditions. We also compared these qPCR methods to traditional bacterial culture experiments using human bile spiked with S. Typhi and antibiotics commonly used during cholecystectomy surgery.

## RESULTS

### Diagnostic targets and probeset validation.

We evaluated probesets that targeted *oriC*, *viaB* (specifically the *tviB* gene), *fliC*-d, STY0201, and *stoD* (Table 1). The *oriC* probeset targets the origin of replication (*oriC*) of S. enterica (but not S. bongori) and is located in the *mioC* gene (9, 10). The *viaB* locus encodes for the biosynthesis of the Vi capsule, which is found in S. Typhi and some serovar Paratyphi C and Dublin strains but not other Salmonella spp. (11, 12). The *fliC*-d probeset detects the gene that encodes type ‘d’ Phase 1 flagellin and therefore will detect S. Typhi and approximately 80 other Salmonella serovars with this Phase 1 flagellin, although S. Typhi is the only one commonly associated with human disease (13, 14). The *stoD* gene (STY1076) has not previously been targeted for a diagnostic. StoD (Salmonella Typhi outer protein D) is a Salmonella Pathogenicity Island 1 (SPI-1) type III secretion system effector containing an active C-terminal RING-type E3 ubiquitin-ligase domain (15). The *stoD* locus is highly conserved among S. Typhi strains. In a Large Scale–BLAST Score Ratio (16) analysis of strains isolated in Chile (17), we found that the *stoD* locus was conserved among S. Typhi isolates but not present in serovars Paratyphi A, Paratyphi B, Typhimurium, Enteritidis, or Newport. In a BLAST search of the NCBI database using the *stoD* gene sequence, there were only two hits to non-S. Typhi genomes: *S.* Newport strain WA_14882 (84% nucleotide identity) and *S.* Paratyphi B Java strain NCTC5706 (99% nucleotide identity).

**TABLE 1:** Primer and probe sequences for target genes

Target		Sequence (5′–3′)	Reference
*oriC*	Forward	AGCCAAATCTCCGCTGGAT	This study
	Reverse	CGGAACTGAAAGGCGCTG	
	Probe	FAM-TGATCTTCAGTGTTTCCCCAACCTGTTTTG-QSY	
STY0201	Forward	CGCGAAGTCAGAGTCGACATAG	(8)
	Reverse	AAGACCTCAACGCCGATCAC	
	Probe	VIC-CATTTGTTCTGGAGCAGGCTGACGG-QSY	
*stoD*	Forward	GGCTGCTAACTCCTGACTGTTATTG	This study
	Reverse	CTACAGACCGAGCCATGTTTAGG	
	Probe	VIC-TAGCGTTTCCCTGCCATTCAATATGACG-QSY	
*viaB*	Forward	GCACCGTTTAACCAACATCAAG	This study
	Reverse	TGTACCTGCGCTGATGATCTG	
	Probe	VIC-TTCAACCGCACAGATCGCCGAACT-QSY	
*fliC*-d	Forward	CTTGGCACAGGTTGATACACTT	(7)
	Reverse	GACATGTTGGAGACTTCGGTT	
	Probe	VIC-TGTCTTCTGCCCGTAGCCGTATCG-QSY	
PhHV	Forward	GGGCGAATCACAGATTGAATC	(8)
	Reverse	GCGGTTCCAAACGTACCAA	
	Probe	ABY-TTTTTATGTGTCCGCCACCATCTGGATC-QSY	

### Specificity of probesets.

Probesets were validated for specificity by qPCR on a panel of bacterial strains (Table 2). This included clinical S. Typhi strains from Africa and South America, as well as clinical and reference strains for other Salmonella serovars and a variety of invasive bacterial species. Most of these strains were included because they are commonly isolated from blood (e.g., K. pneumoniae, S. pneumoniae, and A. baumannii) whereas others were included because they have been known to possess the gene targets under consideration (e.g., C. freundii can possess Vi). The STY0201, *fliC*-d, and *sto*D probesets were specific for S. Typhi. The Vi probeset also detected the *tviB* gene (*viaB* operon) from *S.* Paratyphi C. The *oriC* probeset was specific for Salmonella spp.

**TABLE 2:** Specificity of S. Typhi diagnostic qPCR^*a*^ probesets

Bacterial species/serovar	No. of strains	*oriC*	STY0201	*stoD*	*fliC*-d	*tviB*
S. Typhi	11	+	+	+	+	+
*S.* Paratyphi A	5	+	−	−	−	−
*S.* Paratyphi B sensu stricto	2	+	−	−	−	−
*S.* Paratyphi B Java	1	+	−	−	−	−
S. Typhimurium ST19	2	+	−	−	−	−
S. Typhimurium ST313	1	+	−	−	−	−
*S.* Newport	1	+	−	−	−	−
S. Choleraesuis	2	+	−	−	−	−
*S.* Dublin^*b*^	1	+	−	−	−	−
S. Enteritidis	1	+	−	−	−	−
*S.* Paratyphi C	1	+	−	−	−	+
Staphylococcus aureus	1	−	−	−	−	−
Neisseria meningitidis	1	−	−	−	−	−
Citrobacter freundii	1	−	−	−	−	−
Pseudomonas aeruginosa	1	−	−	−	−	−
Klebsiella pneumoniae	1	−	−	−	−	−
Escherichia coli	8	−	−	−	−	−
Enterobacter cloacae	1	−	−	−	−	−
Enterococcus faecalis	1	−	−	−	−	−
Streptococcus pneumoniae	4	−	−	−	−	−
Acinetobacter baumannii	1	−	−	−	−	−

*^a^*qPCR, quantitative real-time PCR.

*^b^*Some clades of *S.* Dublin are Vi positive. They were not tested in this study.

### Limit of detection of probesets using purified DNA.

**FIG 1:**
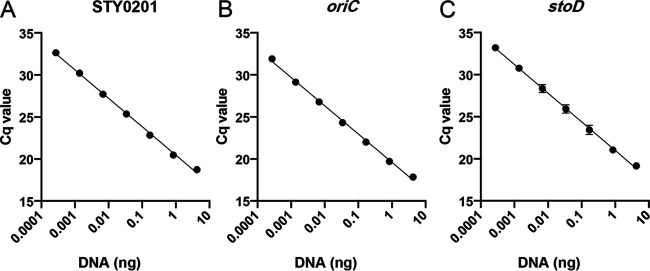
Sensitivity of quantitative real-time PCR (qPCR) probesets with purified genomic DNA. Probesets were tested for sensitivity using decreasing concentrations of purified S. Typhi Ty2 gDNA. (A) STY0201. (B) *oriC*. (C) *stoD*. Data are shown as means ± standard deviations (SDs) from three biological replicates. Cq, quantification cycle.

The *oriC* probeset, which detects all Salmonella, and the two most S. Typhi-specific probesets, STY0201 and *stoD*, were chosen for further analysis. The STY0201, *stoD*, and *oriC* probesets were validated by real-time PCR using S. Typhi Ty2 genomic DNA (gDNA). Linear regression analysis was done on each probeset by using a range of template concentrations from 0.0002 pg to 4 ng of template DNA (Fig. 1). The theoretical limit of detection for each probeset was determined from the line of best fit using a quantification cycle (Cq) cutoff of 35. The *oriC* probeset was the most sensitive and efficient, with a qPCR efficiency of 98.27% and a limit of detection of 0.026 pg of DNA. STY0201 was the next best probeset, with an efficiency of 97.75% and a detection limit of 0.050 pg, while the *stoD* probeset was least efficient at 96.69% and a detection limit of 0.076 pg of DNA.

### Limit of detection of probesets on spiked human bile samples.

DNA extraction was completed on multiple individual human bile samples spiked with 10^2^–10^6^ CFU mL^−1^ of S. Typhi. Genomic DNA was extracted from spiked bile samples by using the QIAamp DNA Blood minikit, with modifications to improve DNA extraction efficacy as described in the Materials and Methods. To decrease the complexity and viscosity of samples, bile aliquots were centrifuged and washed with PBS before extraction. Without washing, sensitivity of the qPCR was severely affected. Increasing the volume of the bile aliquot for DNA extraction, increasing the number of washes, and increasing the amount of DNA template used in the qPCR did not increase qPCR sensitivity (supplemental Table S1).

**FIG 2:**
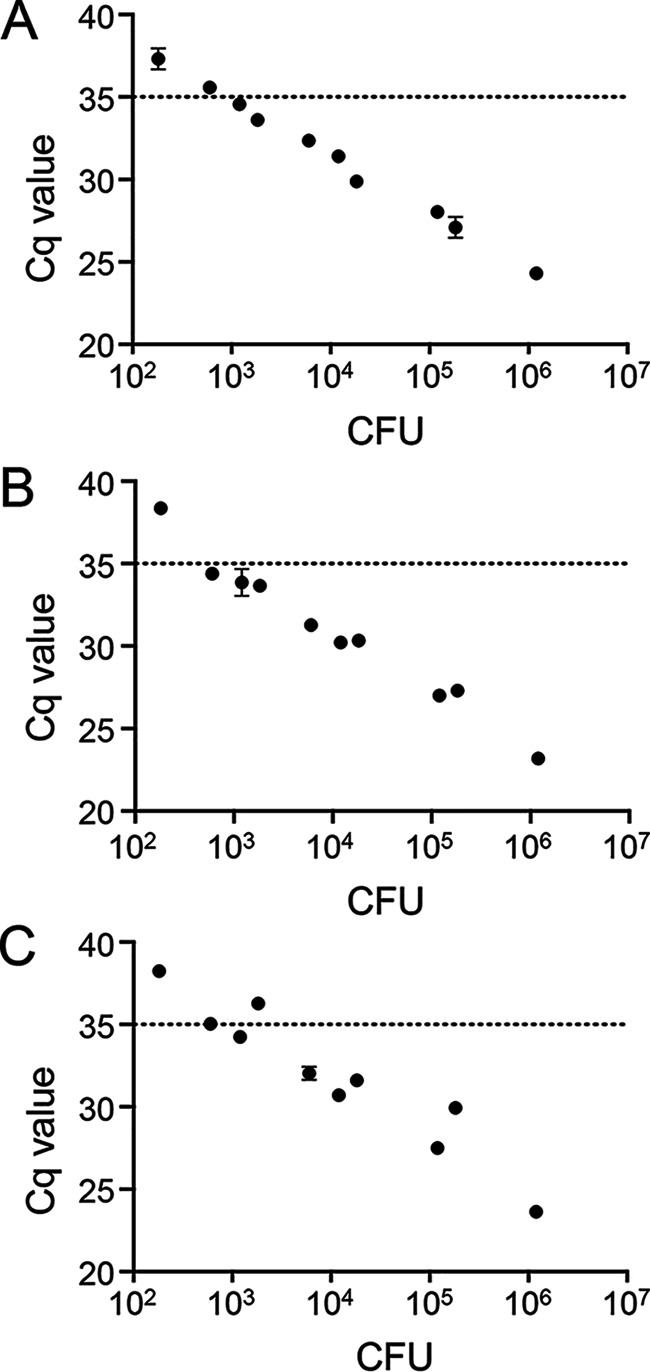
Sensitivity of qPCR probesets on DNA extracted from spiked human bile samples. Human bile samples were spiked with varying concentrations of S. Typhi strain Ty2. DNA was extracted from bile samples using the optimized protocol, and qPCR was completed using either the *oriC* probeset (A), the STY0201 probeset (B), or the *stoD* probeset (C). Data are shown as means ± SDs from duplicate technical replicates.

By linear regression analysis, we determined that by using a cutoff Cq value of 35, the *oriC* probeset could theoretically detect 7.4 × 10^2^ CFU mL^−1^ S. Typhi (Fig. 2A). For the STY0201 probeset, the limit of detection was determined to be 8.5 × 10^2^ CFU mL^−1^ (Fig. 2B), while for *stoD*, the theoretical limit of detection was 1.2 × 10^3^ CFU mL^−1^ (Fig. 2C).

The probesets were designed to be multiplexed, such that the phocine herpesvirus (PhHV) control, *oriC*, and either STY0201 or *stoD* could be tested in a single reaction. However, when this was tested on DNA extracted from spiked bile, the detection limit for *oriC* in a duplex reaction with PhHV increased more than 2 logs. The combination of probesets within one reaction therefore significantly decreased the sensitivity of the assay.

### Reproducibility of detection in bile.

**FIG 3:**
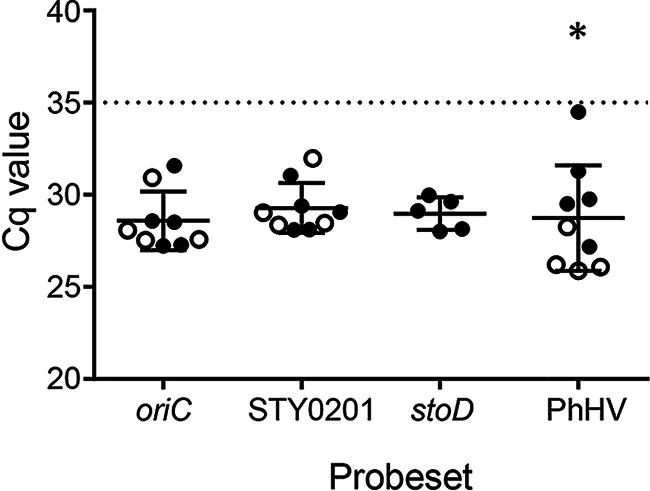
Variability in qPCR sensitivity between human bile samples. Bile samples from human subjects in the United States (filled circles) or Chile (open circles) were spiked with 10^5^ CFU mL^−1^ of S. Typhi. Quantitative PCR was run on DNA extracted from spiked samples in duplicate using each of the four test probesets. Data are shown as means ± SDs; *, *P* < 0.05.

To evaluate reproducibility, we performed qPCR on bile samples from five donors in the United States and four patients in Santiago, Chile. Samples were spiked with 10^5^ CFU mL^−1^ of S. Typhi and then subjected to the optimized DNA extraction and qPCR in duplicate wells. The distribution of Cq values is shown in Fig. 3. The level of variation seen with the STY0201 and *oriC* probesets was roughly equivalent, with Cq values ranging from 28.09 to 31.98 and 27.23 to 31.57, respectively. There was no significant difference in sensitivity between the bile from these two locations for each of these Salmonella-specific probesets. The largest variation was seen with the PhHV probeset, which ranged from 25.88 to 34.49. For this probeset, the sensitivity was significantly different between locations, with the samples tested in the United States having a higher average Cq. The *stoD* PCR produced very consistent Cq values for the samples from the United States (range: 28.00–29.98) but was not tested on the samples from Chile.

### Detection of S. Typhi in antibiotic-spiked bile by culture and qPCR.

To determine the effect of antibiotics on detection of S. Typhi in bile by either culture or qPCR, we carried out several spiking experiments. Bile samples were spiked with S. Typhi and one of three cephalosporins, cefazolin, cefotaxime, or ceftriaxone, that are commonly administered parenterally to patients just prior to undergoing cholecystectomy to diminish surgical wound infections (18). Samples were then processed for qPCR or cultured in broth.

The minimum inhibitory concentration (MIC) for each antibiotic was first tested in selenite broth and found to be 5 μg mL^−1^ for cefazolin, 1.25 μg mL^−1^ for cefotaxime, and 1 μg mL^−1^ for ceftriaxone. We hypothesized that the dilution of antibiotic spiked bile in broth would permit growth of the bacteria. Culture samples were inoculated into selenite broth using dilutions of 1 in 50, 1 in 100, 1 in 200, and 1 in 500. S. Typhi could be cultured from cefazolin-spiked bile down to a concentration of 10^2^ CFU mL^−1^, using a dilution of 1 in 100 (Table 3). Above a concentration of 10^3^ CFU mL^−1^, a 1 in 50 dilution of cefazolin spiked bile was sufficient for growth. In contrast to cefazolin, no dilution (up to 1 in 500) of cefotaxime or ceftriaxone spiked bile was sufficient to permit growth, up to a bacterial concentration of 10^5^ CFU mL^−1^.

**TABLE 3:** Detection of S. Typhi from antibiotic spiked bile by culture and qPCR using the STY0201 probeset

	Cephalosporin (50 μg mL^−1^)^*a*^					
Concn of S. Typhi	Cefazolin		Cefotaxime		Ceftriaxone	
	Culture (dilution)	qPCR (Cq)^*b*^	Culture	qPCR (Cq)	Culture	qPCR (Cq)
10^2^ CFU mL^−1^	+ (1 in 100)	37.0	ND^*c*^	37.3	ND	38.1
10^3^ CFU mL^−1^	+ (1 in 50)	33.1	−	34.1	−	33.5
10^4^ CFU mL^−1^	+ (1 in 50)	30.3	−	30.5	−	30.5
10^5^ CFU mL^−1^	ND	ND	−	26.7	−	26.7

*^a^*+, growth; −, no growth.

*^b^*Cq, quantification cycle.

*^c^*ND, not determined.

Detection of S. Typhi by qPCR was not affected by the inclusion of antibiotics, regardless of which cephalosporin was used. The Cq value for the STY0201 probeset is shown for each dilution in each antibiotic (Table 3). The *oriC* and *stoD* probesets showed similar results. Under these conditions, the qPCR was more sensitive than culture in the presence of cefotaxime and ceftriaxone and equally as sensitive in the presence of cefazolin.

## DISCUSSION

One of the major issues affecting the typhoid field is a lack of accurate epidemiological data quantifying the prevalence of chronic typhoid carriers in disparate venues representing different levels of typhoid endemicity. This is in part related to the lack of appropriate diagnostics. In this study, we developed a real-time PCR assay to detect S. Typhi in bile samples from chronic carriers as an adjunct to culture methods, particularly in patients who may have received antibiotics shortly prior to obtaining the bile specimen. Of the four S. Typhi-specific probesets tested, two (STY0201 and *stoD*) were highly specific for S. Typhi. While the *fliC*-d probeset was also S. Typhi specific within the 48 strains comprising our bacterial panel, *fliC*-d is present in more than 80 other serovars that we did not test. As expected, the *tviB* (part of *viaB*, which ecodes Vi) probeset detected *S.* Paratyphi C as well as S. Typhi. Additionally, the genes that encode Vi are located on a mobile genetic element and can therefore be lost from the bacterium; *tviB* may not be a robust diagnostic marker (12, 19).

Several groups have developed qPCR-based assays to detect S. Typhi in blood (7–9, 20), stool (21), or both (22). However, as far as we are aware, we are the first to develop a qPCR-based method intended to be used as a diagnostic to detect S. Typhi in bile. In humans, the average concentration of S. Typhi in bile isolated from patients in Nepal (who did not receive antibiotics prior to cholecystectomy) was 5.2 × 10^4^ CFU mL^−1^ (6). Our qPCR method was highly sensitive in human bile samples, with a limit of detection of between 10^2^ and 10^3^ CFU mL^−1^ for each probeset. The published STY0201 probeset (8) was slightly more sensitive than the novel *stoD* probeset. Although we do not yet have clinical data on the sensitivity of these qPCR assays in patients, we would predict that all the probesets would be sufficiently sensitive to detect S. Typhi in the majority of chronic carriers. Determining the diagnostic sensitivity of this approach will require further testing in typhoid endemic regions. Additionally, routine use of the qPCR method to monitor chronic carriers will require further validation (23).

In addition to sensitivity, the assay was robust when tested in reconstruction experiments using a range of human samples in both the United States and Chile. Bile consistency can vary widely among individuals, leading to some variation in DNA extraction efficacy. The assay was, however, able to deal with this variation on a small subset of volunteers. The PhHV probeset is most sensitive to changes in DNA extraction efficiency due to the very small amount of plasmid DNA spiked into bile samples to act as the extraction control. This most likely explains the significant difference in sensitivity between the samples tested in the United States and Chile, as one bile sample in the United States produced high Cq values. Although increasing the amount of PhHV template plasmid would improve this variation between samples, the plasmid also decreased sensitivity for the other probesets, so plasmid concentration was kept to a minimum. Unfortunately, we were unable to obtain matching sensitivity data for the *stoD* probeset, as the Chile bile reconstruction data were generated during method transfer to the Instituto de Salud Publica in Santiago for a typhoid carrier study and the *stoD* probeset was not used in that study.

The probesets were originally designed to allow for multiplexing of the samples, with the probes for PhHV, *oriC*, and STY0201/*stoD* each carrying different fluorophores. However, the combination of two or more probes in a single qPCR decreased the sensitivity of each probe. In cases where high bacterial counts are expected and sensitivity does not need to be maximized, these probesets could be combined in a multiplexed reaction. If sensitivity was of paramount importance, then we would suggest that *oriC* and PhHV reactions be done on every sample, with those being positive for *oriC* being tested further with either STY0201 or *stoD*.

If the bile specimen is collected from a patient at the time of cholecystectomy, one of the major hurdles to detecting S. Typhi in a putative chronic carrier is the presence of antibiotics, since parenteral antibiotics, particularly cephalosporins, prior to surgery to prevent wound infections have become widely practiced standard of care. Morever, these antibiotics are concentrated in bile. In Chile, the most common drug given to patients undergoing elective cholecystectomy is cefazolin. Ceftriaxone, cefotaxime, and other parenteral antimicrobials are normally used pre- and postsurgery in patients with acute cholecystitis undergoing emergency operation. The penetrance of each of these antibiotics into the gallbladder differs, with levels in bile judged in some studies to be approximately five times that in serum for ceftriaxone, three times for cefazolin, and only 0.75 times for cefotaxime (24). From the literature, a 500-mg intramuscular dose of cefazolin produced a biliary antibiotic concentration of 32.8 μg mL^−1^ after one dose, rising to 92.1 μg mL^−1^ after four doses over 24 h (25). In another study, seven doses of cefotaxime (1 g intravenous) gave a bile concentration of 33.7– 82.4 μg mL^−1^ (26). Two other studies on cefotaxime using a single 2-g intravenous dose elicited bile concentrations of 22 μg mL^−1^ and 87.6 μg mL^−1^ (27–29). In contrast, concentrations of ceftriaxone in bile after a 1 g dose were found to be around 250 μg mL^−1^ (27, 30).

In our antibiotic spiking experiments, we chose a conservative starting bile concentration of 50 μg mL^−1^. Cefazolin could be sufficiently diluted out in selenite broth to allow detection of S. Typhi by culture, whereas no amount of dilution could rescue samples containing cefotaxime or ceftriaxone. From the MIC, we would expect that dilutions of greater than 1 in 100 should theoretically permit growth. However, the combination of antibiotic potency, bacterial concentration, and bile associated factors do not make this possible. In contrast, the qPCR detection method was not affected by antibiotic presence. Presumably, this is due to the qPCR not being dependent on live bacteria. These results are highly dependent on the final antibiotic concentration that is reached in human bile after administration of antibiotics prior to surgery. As mentioned above, this value is difficult to predict and will depend on numerous factors including antibiotic dose, dosage route, time between doses, antimicrobial resistance pattern of the bacteria, and the presence of biliary tract obstructions.

When comparing the results of the qPCR and culture methods, the theoretical limit of detection of S. Typhi in the presence of cefazolin was approximately equal. However, in the presence of cefotaxime or ceftriaxone, the qPCR was superior. As such, the qPCR method should be the preferred choice for S. Typhi detection in bile obtained from patients undergoing cholecystectomy and where cephalosporins other than cefazolin are administered prophylactically to prevent postoperative infections. We also fully recognize that in situations where bile or bile-containing duodenal fluid specimens can be obtained directly for culture by use of string tests, intestinal tubes, or endoscopy, S. Typhi can be readily cultured in large numbers from chronic or short-term infections and the qPCR is not critical. On the other hand, even with these specimen collection methods qPCR will be an important tool if those patients are receiving antibiotics.

With the recommended deployment of new conjugate vaccines for S. Typhi, a new tool is being introduced that has the potential to reduce host susceptibility leading to a fall in incidence rates of typhoid fever in endemic areas. If the typhoid burden drops markedly as a result of vaccination, there will be interest in some locales in attempting to detect chronic carriers, as these individuals will constitute the silent long-term reservoir of S. Typhi. These carriers will be a potential source of residual short-cycle transmission to susceptible persons if their personal hygiene practices are inadequate and they contaminate food that they handle. In areas where endemic S. Typhi remain susceptible to antibiotics capable of eliminating chronic gallbladder carriage, identified chronic carriers can be treated to shrink the long-term reservoir of S. Typhi (31).

In conclusion, the optimization of a qPCR detection method for S. Typhi chronic carriage may overcome issues associated with antibiotic presence in bile samples and would therefore be superior over culture. The use of this qPCR in both typhoid endemic and formerly endemic regions can provide us more information about the levels of chronic typhoid carriage among the population and will guide future efforts in typhoid control and eradication.

## MATERIALS AND METHODS

### Ethics statement.

Bile samples were collected from patients undergoing cholecystectomy in Santiago, Chile. Samples were completely de-identified, and no demographic or other identifiable information was taken from patients. This was determined to be Not Human Subjects Research by the Institutional Review Board at the University of Maryland, Baltimore (HP-00081332).

### Bacterial strains and genomic DNA.

Bacterial strains used in this study consisted of both prototypic and clinical strains and are listed in Table 4. Bacteria were grown under aerobic conditions at 37°C. S. enterica, E. coli, P. aeruginosa, K. pneumoniae, and C. freundii strains were grown on Hy-Soy medium (0.5% wt/vol Hy-yest [Kerry Biosciences, Beloit, WI], 1% wt/vol Soytone [TEKNova, Hollister, CA], and 0.5% wt/vol NaCl [American Bio, Natick, MA]). E. cloacae and E. faecalis were grown on Bacto Tryptic Soy Agar (BD, Sparks, MD). S. aureus, S. pneumoniae, and N. meningitidis were grown on sheep’s blood agar plates (Tryptic soy agar, 5% vol/vol sheep blood [Waltz Farm, Smithsburg, MD]), with N. meningitidis incubated with 5% CO_2_. Genomic DNA was isolated using the Bacterial Genomic DNA isolation kit (Norgen Biotek Corp., Thorold, Canada). A. baumannii genomic DNA (gDNA) was a gift from D. Rasko at the Institute for Genome Sciences, University of Maryland, Baltimore. Bile spiking experiments were performed with S. Typhi strains Ty2 and ISP1820.

**TABLE 4:** Bacterial strains used in this study

Bacterial species/serovar	Strain ID	Details	Reference
S. Typhi	Ty2	Reference strain	(32, 33)
	CDC 06-0418	Reference strain	CDC^*a*^
	704223	Clinical isolate (Pakistan)	(34)
	503879	Clinical isolate (India)	(34)
	704085	Clinical isolate (Pakistan)	(34)
	I56	Clinical isolate (Mali)	(35)
	S14	Clinical isolate (Mali)	(35)
	6494 EBC	Clinical isolate (Chile)	(36, 37)
	1277 MBM	Clinical isolate (Chile)	(36, 37)
	POV 7840	Clinical isolate (Chile)	(36, 37)
	ABL 114	Clinical isolate (Chile)	(36, 37)
*S.* Paratyphi A	ATCC 9150	Reference strain	ATCC^*b*^
	ACE 59	Clinical isolate (Chile)	(36, 37)
	AOS 5	Clinical isolate (Chile)	(36, 37)
	BHR 6	Clinical isolate (Chile)	(36, 37)
	VOM 6681	Clinical isolate (Chile)	(36, 37)
*S.* Paratyphi B sensu stricto	CMF 6999	Clinical isolate (Chile)	(36, 37)
	ELB 6380	Clinical isolate (Chile)	(36, 37)
	BAS 9	Clinical isolate (Chile)	(36, 37)
	6198	Clinical isolate (Chile)	(36, 37)
*S.* Paratyphi B Java	CDC 00-0301	Reference strain	CDC
	CDC 03-0451	Reference strain	CDC
	CDC 01-0516	Reference strain	CDC
S. Typhimurium	SL1344	Reference strain	(38)
	LT2	Reference strain	(39)
	D23580	Clinical isolate (Malawi)	(40)
*S.* Newport	CDC 07-0044	Reference strain	CDC
S. Choleraesuis	CDC 06-0868	Reference strain	CDC
	CDC 06-0894	Reference strain	CDC
*S.* Dublin	CDC 06-0707	Reference strain	CDC
S. Enteritidis	R11	Clinical isolate (Mali)	(35)
*S.* Paratyphi C	CDC 32	Reference strain	CDC
S. Typhi	I8	Clinical isolate (Mali)	(35)
S. aureus	ATCC 29213	Reference strain	ATCC
N. meningitidis	539	CVD culture collection	A. Cross
C. freundii	1051	Clinical isolate	CVD culture collection
P. aeruginosa	4411116	Clinical isolate (Mali)	S. Sow
K. pneumoniae	15AP506917	Clinical isolate (Sweden)	A. Brauner
E. coli	DH5a	Laboratory strain	(41)
	SMS-3-5	Environmental	(42)
	15-02699	Invasive clinical isolate (USA)	J. Johnson
	16-02043	Invasive clinical isolate (USA)	J. Johnson
	16-04232	Invasive clinical isolate (USA)	J. Johnson
	13145/3	Invasive clinical isolate (DRC)	J. Jacobs
	13575/3	Invasive clinical isolate (DRC)	J. Jacobs
	EDL933	EHEC; Reference strain	(43)
E. cloacae	6013	CVD culture collection	Unknown
E. faecalis	ATCC 29212	Reference strain	ATCC
S. pneumoniae	6b	CVD culture collection	A. Cross
	14	CVD culture collection	A. Cross
	19F	CVD culture collection	A. Cross
	23	CVD culture collection	A. Cross
A. baumannii	268860	Reference strain	D. Rasko

*^a^*CDC, US Centers for Disease Control and Prevention.

*^b^*ATCC, American Type Culture Collection.

### Target sequences and primer/probe design.

In order to design a probset specific for *stoD* (STY1076), we first performed a BLAST search (performed 27 December 2016) of the NCBI nucleotide database using the *stoD* gene sequence. Due to shared nucleotide identity with *S.* Newport and *S.* Paratyphi B Java, we designed the probe such that it would bind to *stoD* from S. Typhi but not serovars Paratyphi B or Newport. Primer and probe sequences are listed in Table 1. Sequences for *oriC*, *stoD*, and *tviB* (*viaB* operon) were designed using Primer Express 3.0.1 (Applied Biosystems, Foster City, CA).

### Quantitative real-time PCR.

Amplification reactions were carried out in a 20 μL total volume using 10 μL TaqMan Universal PCR Master Mix (Thermo Scientific, Waltham, MA), 1 μL probemix (250 nmol l^−1^ probe and 300 nmol l^−1^ each primer), and 2 μL of DNA template. Reactions were run on the 7500 Fast Dx Real-time PCR machine (Applied Biosystems), using the default cycling conditions (initial denaturation at 95°C for 20 s and then 40 cycles of annealing at 95°C for 3 s and extension at 60°C for 30 s). Reactions that reached threshold within 35 cycles (Cq < 35) were recorded as positive unless otherwise specified. Sensitivity of the probesets was determined by using genomic DNA and taking the average of three independent experiments.

### DNA extraction from spiked bile samples.

Human bile was purchased from Cedarlane (Burlington, NC) or obtained from patients undergoing cholecystectomy in Santiago, Chile. Bile samples (400 μL) were spiked with S. Typhi at concentrations from 10^2^ to 10^7^ CFU mL^−1^. DNA extractions were completed using the QIAamp DNA minikit (Qiagen, Hilden, Germany) using the method for extraction from tissue samples, with the following modifications. Prior to DNA extraction, bile was pretreated by washing in PBS. To wash, samples were centrifuged for 5 min at 13,000 × *g*, resuspended in 1 mL PBS, and vortexed well. Samples were then centrifuged again, the supernatant removed, and the pellet resuspended in 200 μL Buffer ATL. Lysis of bile samples was achieved by adding 50 μL of proteinase K (Qiagen) and incubating for 45 min at 56°C. The volumes of Buffer AL and ethanol were each increased from 200 to 250 μL. Directly after the addition of Buffer AL, each sample was spiked with 5 μL of 100 pg μL^−1^ plasmid DNA containing the PhHV target sequence gene gB, which served as an internal positive control for DNA extraction (8, 9). Samples were eluted from the column in 100 μL of Buffer AE, which was then returned to the column and the elution repeated to increase recovery.

### Detection of S. Typhi in antibiotic-spiked bile samples.

Human bile samples were spiked with 1–2 × 10^2^ to 10^5^ CFU mL^−1^ of S. Typhi (as described above), and 50 μg mL^−1^ of either cefazolin, cefotaxime, or ceftriaxone. Samples were left on ice for 10–30 min before detection by qPCR (as above) or culture. For culture, bile samples were diluted in Difco Selenite Cystine broth (BD) using dilutions from 1 in 50 to 1 in 500 and cultured at 37°C with shaking for 24–48 h. For confirmation and quantitation of S. Typhi, samples were plated on BD Salmonella-Shigella agar (BD) or Remel Bismuth-Sulfite agar (Thermo Scientific).

### Data analysis.

Linear regression analysis was performed using GraphPad Prism 6. Students *t* test was used for comparison of probeset sensitivity in bile from United States versus Chile.

### Data availability.

All relevant data are included in this manuscript.
