# Supplementary material for: Detection of Salmonella Typhi in Bile by Quantitative Real-Time PCR
Source: Microbiol Spectr. 2022 May 31;10(3):e00249-22. doi: 10.1128/spectrum.00249-22 (PMC9241738; doi:10.1128/spectrum.00249-22)
Supplement: SUPPLEMENTAL FILE 1 — Supplemental material. Download spectrum.00249-22-s0001.pdf, PDF file, 0.02 MB [file spectrum.00249-22-s0001.pdf]

Supplementary table. Optimization of bile processing conditions.

			Average Cq for designated probesets and volume of template						
			oriC			STY0201			
Volume of bile	Spiked with *S.* Typhi Ty2 CFU	No. of washes of bile prior to DNA extraction	2 µl	4 µl	6 µl	2 µl	4 µl	6 µl	Pass/Fail
200 µl	10^4	1X	35.37	NT[a]	Undet.[b]	Undet.	NT	Undet.	Fail
400 µl	10^4	1X	31.41	31.73	32.27	32.02	32.25	36.20	Pass
	10^3	1X	29.14	NT	29.21	30.42	NT	31.14	Pass
	10^3	2X	28.08	NT	27.92	29.17	NT	28.99	Pass
500 µl	10^4	1X	31.26	Undet.	Undet.	32.01	34.76	Undet.	Fail
1 ml	10^4	3X	36.39	37.41	Undet.	36.39	37.01	Undet.	Fail

[a], Not tested

[b], Cq undetermined
